# Case Report: Clinical Outcome and Image Response of Two Patients With Secondary High-Grade Glioma Treated With Chemoradiation, PCV, and Cannabidiol

**DOI:** 10.3389/fonc.2018.00643

**Published:** 2019-01-18

**Authors:** Paula B. Dall'Stella, Marcos F. L. Docema, Marcos V. C. Maldaun, Olavo Feher, Carmen L. P. Lancellotti

**Affiliations:** ^1^Department of Neuro-Oncology, Sirio Libanes Hospital, São Paulo, Brazil; ^2^Department of Pathology, Santa Casa de São Paulo, São Paulo, Brazil

**Keywords:** high-grade glioma, cannabidiol, pseudoprogression, PCV, chemoradiation, cancer, cannabis, THC-tetrahydrocannabinol

## Abstract

We describe two patients with a confirmed diagnosis of high-grade gliomas (grades III/IV), both presenting with O6-methylguanine-DNA methyltransferase (MGMT) methylated and isocitrate dehydrogenase (IDH-1) mutated who, after subtotal resection, were submitted to chemoradiation and followed by PCV, a multiple drug regimen (procarbazine, lomustine, and vincristine) associated with cannabidiol (CBD). Both patients presented with satisfactory clinical and imaging responses at periodic evaluations. Immediately after chemoradiation therapy, one of the patients presented with an exacerbated and precocious pseudoprogression (PSD) assessed by magnetic resonance imaging (MRI), which was resolved in a short period. The other patient presented with a marked remission of altered areas compared with the post-operative scans as assessed by MRI. Such aspects are not commonly observed in patients only treated with conventional modalities. This observation might highlight the potential effect of CBD to increase PSD or improve chemoradiation responses that impact survival. Further investigation with more patients and critical molecular analyses should be performed.

## Background

Gliomas, the most common primary brain tumors, account for more than 40% of all CNS neoplasms and are highly resistant to available therapeutic approaches (1, 2). These tumors often have a poor prognosis with a median survival time of ~1 year for patients with high-grade gliomas (grades III/IV) (3, 4). The Stupp protocol has become the standard of care treatment for primary glioblastoma (GBM), and has led to significantly improved survival (5). It consists of chemoradiation (combining chemotherapy and radiation therapy simultaneously) followed by adjuvant temozolomide (TMZ), an alkylating agent, and is associated with a medium survival rate of 15 months.

It was reported that 10% of GBM are secondary (progressing from a low-grade tumor, IDH mutated) (6) and that these patients often previously received prior chemotherapeutic treatment, there being no stand-by treatment for this condition. After progression from a low-grade tumor to a high-grade tumor, combination radiotherapy with TMZ is a good therapeutic option (5). However, patients who failed with TMZ started PCV, a multiple drug regimen (procarbazine, lomustine, and vincristine) adjuvant. Because of the adverse events (nausea and seizures) related to this treatment, we decided to use cannabidiol (CBD) from the irradiation phase onwards. A significant inflammatory response to the therapy was observed in one patient, which we considered pseudoprogression (PSD), and in the other patient, it presented with a marked remission of altered areas compared with the post-operative scans as assessed by MRI.

Tumor pseudoprogression (PSD) can occur in up to 30% of patients after chemoradiation, especially O6-methylguanine-DNA methyltransferase (MGTM) methylated cases. This corresponds to a treatment-related increase in lesion size related to inflammatory responses that simulate the progression of the disease. In almost 60% of cases, PSD occurs within the first 3–6 months after completing chemoradiation (5, 7, 8). PSD does not represent a progression of the disease, and is often a marker of longer survival, presumably because it represents a robust response to treatment (9).

Cannabidiol (CBD) is a prevalent natural cannabinoid. It is a non-intoxicating compound, instead of which may have, antipsychotic effects (10–12), and promote a wide spectrum of pharmacological effects including anti-inflammatory, anti-oxidant, anti-proliferative, anti-invasive, anti-metastatic, and pro-apoptotic activity (13–15). Recent studies have suggested CBD has immunomodulatory effects (15–18).

Cannabinoids are becoming promising anti-tumor drugs and increasing preclinical evidence reported that these compounds, including Δ^9^-tetrahydrocannabinol (THC), inhibited tumor growth in animal models of cancer by targeting specific cancer-cell signaling pathways. Furthermore THC act as broad-spectrum antiemetic against different emetic stimuli. And interestingly displayed as an active agent against immediate and delayed phases of chemotherapy-induced nausea and vomiting (19–23). Unfortunately, there are very few reports of the potential anti-tumor activity of cannabinoids in cancer patients.

Here, we describe two patients with a confirmed diagnosis of high-grade gliomas (grades III/IV), both presenting with MGMT methylated and IDH-1 mutated who, after subtotal resection, were submitted for chemoradiation followed by PCV associated with CBD. Despite the impressive inflammatory imaging immediately after chemoradiation, both patients presented with satisfactory clinical and imaging responses in the following periodic evaluations.

The dose of CBD administered was based on individual tolerance and side effects (mainly drowsiness). All patients received it orally, as capsules containing 50 mg of CBD [CBDRx Functional Medicine Company, who confirmed it is made by hemp oil that contains < 0.3% of tetrahydrocannabinol (THC)]. This regimen was maintained during the whole study, which lasted ~2 years after the second surgery. It is important to mention that, in order to alleviate the symptoms of chemotherapy, both patients used vaporisers to inhale THC-rich cannabis flowers during their first year of treatment.

## Case Presentation

Patient 1 was a 38 years-old male. In May 2010, this patient was diagnosed with glioma soon after an episode of seizures. MRI showed intra-axial expansive and infiltrative lesions that were cortical and subcortical, and which affected the anterior half of the right temporal lobe and extending from the pole to the Sylvian fissure superiorly and to the right parahippocampal gyrus, posteriorly, and medially. Partial surgical resection was performed in August 2010 and the first pathologic diagnosis was astrocytoma grade II. He underwent chemotherapy with TMZ at a dose of 2,000 mg with cycles every 28 days for 5 days in the years 2011–2013, with no tumor regrowth until the beginning of 2015. At this time, he underwent MRI, which was used to compare the discrete extension of the signal alteration areas, especially the subinsular regions. In March 2015, he resumed chemotherapy with TMZ at a dose of 100 mg/day and the patient then lost 12 kg of body weight, which was associated with anorexia, insomnia, and depression. In May 2015, he suffered a seizure requiring hospitalization. In June 2015, the patient resumed the old chemotherapy regimen with TMZ (2,000 mg every 28 days for 5 days), and a follow-up with MRI; however, the tumor size continued to increase. In January 2016, the neuro-oncology team decided to discontinue treatment with TMZ considering the risk/benefit and planned a surgical re-approach. This was followed by chemoradiation and lasting 6 cycles of PCV associated with CBD. The CBD dosage was ranging from 300 to 450 mg/day.

During chemoradiation, the patient had an excellent clinical performance, practiced sports and had few symptoms of fatigue and/or nausea.

**Figure 1:**
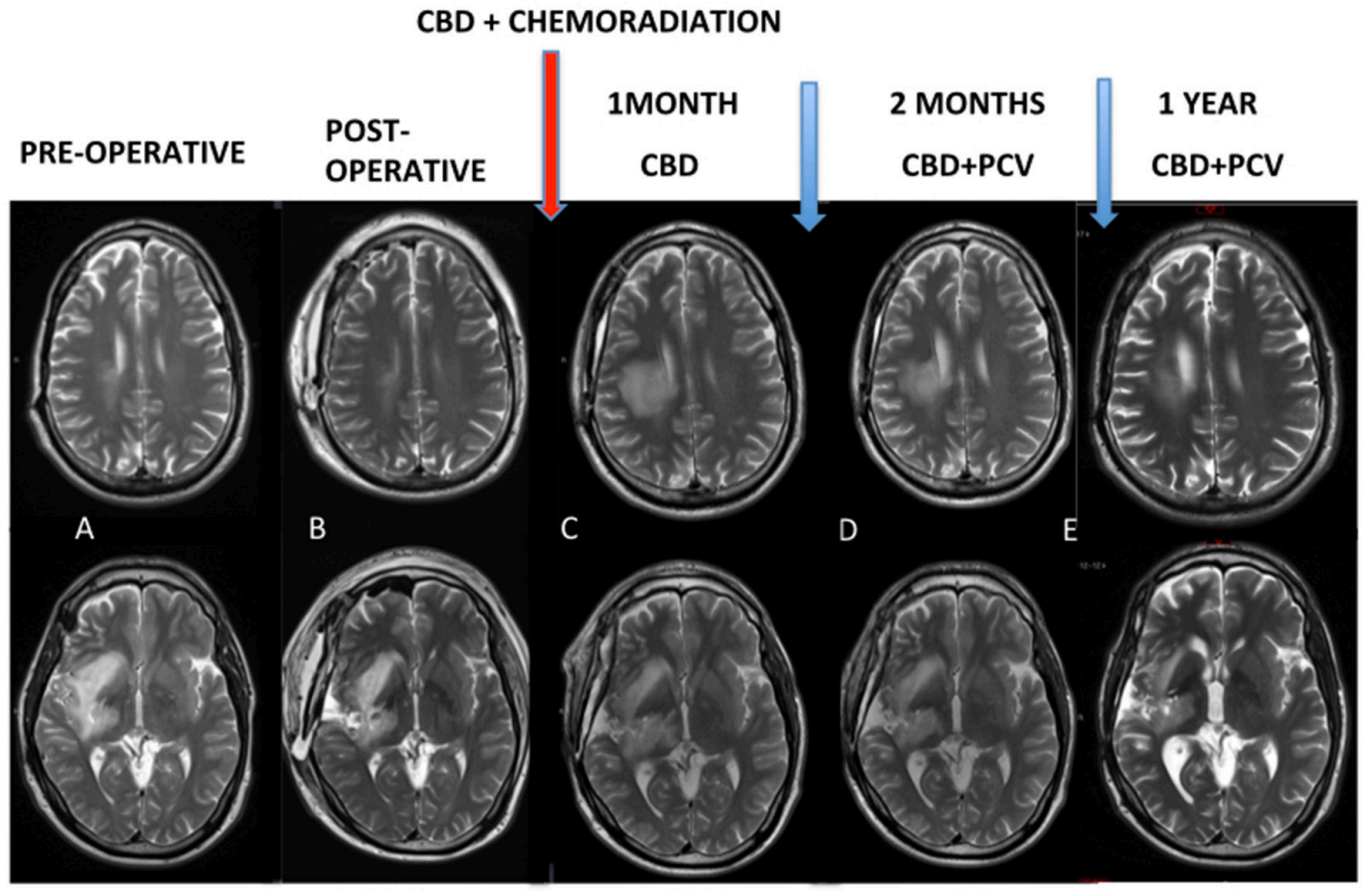
**(A,B)** Pre and post-operative MRI. Red arrow: chemoradiation associated with cannabidiol in a dosage ranging from 300 to 450 mg/day. **(C)** Control MRI after 1 month of the end of the chemoradiation was characterized an exacerbated and ultra-precocious phenomenon of pseudoprogression with increased edema and inflammatory disease. **(D,E)** MRI controls demonstrated progressive reduction of these findings.

At 1 month after the end of chemoradiation, control MRI (Figure 1) was characterized by exacerbation and the ultra-precocious phenomenon of PSD with increased edema and inflammatory disease characterized by extensive areas of contrast enhancement associated with tissue hypoperfusion (not shown). MRI controls demonstrated the progressive reduction of these findings.

**Figure 2:**
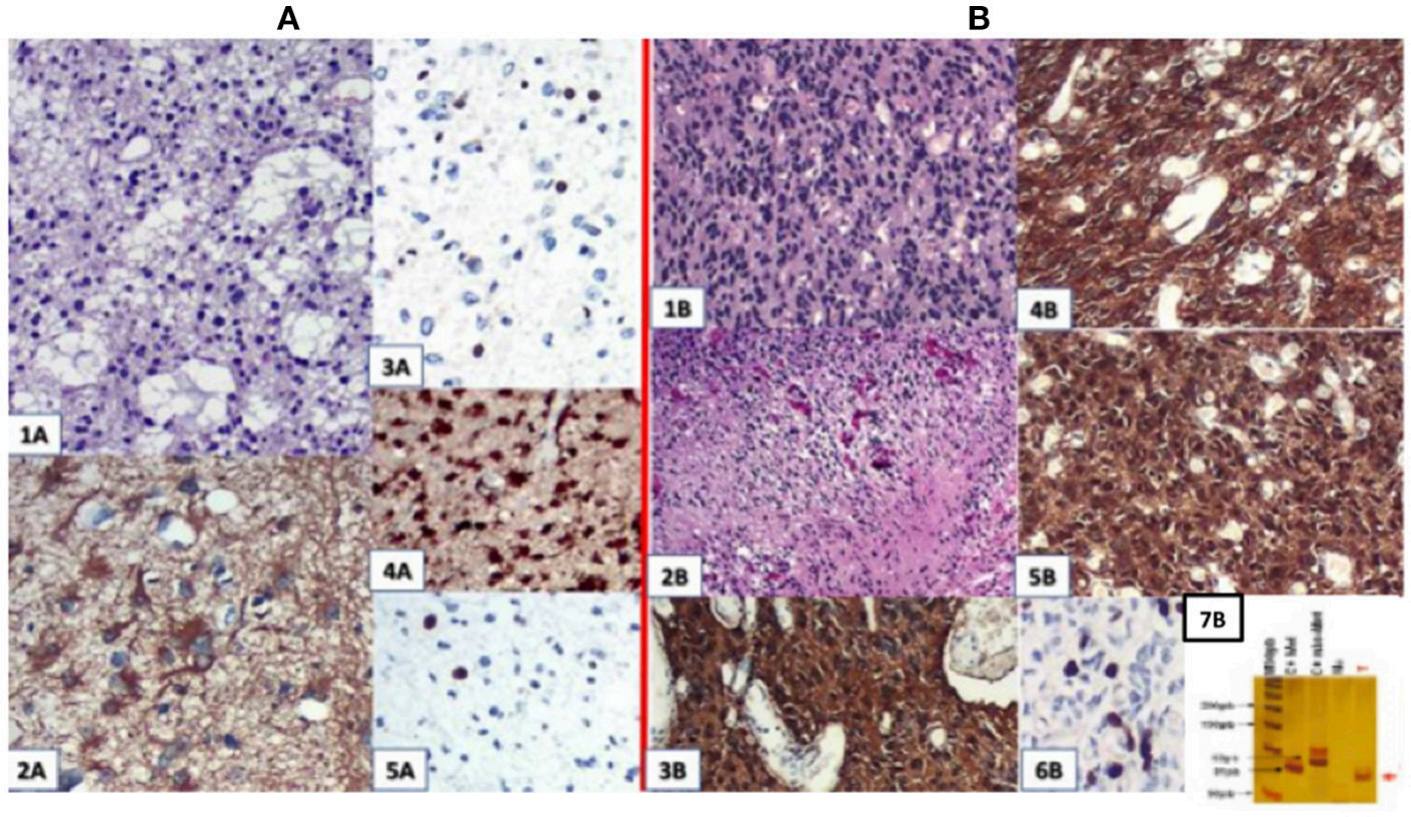
**(A)** First surgery: June/2010−1A.HE; 2A. GFAP; 3A. ATRX; 4A. IDH-1 ; 5A.Ki67:4,5%. **(B)** Second surgery: May/2016−1 B and 2 B (micronecrosis). HE; 3B. GFAP; 4B. EGFR; 5B. IDH-1 ; 6B. Ki67: 30%; 7B: MGMT methylated.

The result of a pathological study after the first surgery was astrocytoma grade II with Ki67 staining of 5%. After the second surgery, he progressed to GBM grade IV (Figure 2), related to increased cellularity, frequent mitosis, presence of micronecrosis, microvascular proliferation/endothelial, Ki67 staining of 30%, and loss of ATRX expression. Biomolecular marker analysis indicated IDH-1 mutated and MGMT methylated.

Patient 2 was a 38 years-old male diagnosed as left temporal glial neoplasia in May 2014 after a seizure. MRI showed an expansive infiltrative lesion predominantly in the subcortical region, with poorly defined contours located in the left temporal lobe, involvement of the upper, middle, and lower temporal gyrus, and an increase in the left temporal gyrus cortex. The lesion compromised a large part of the temporal lobe and extended to the temporal isthmus, the posterior aspect of the insula, and was deep in the trigeminal effigy of the left lateral ventricle. There was diffuse erasure of the regional cortical sulci and the Sylvian fissure, as well as a slight compression over the atrium of the left lateral ventricle. Stereotactic biopsy on April 2014 indicated a diagnosis of oligodendroglioma grade II. He received TMZ 1,875 mg with cycles every 23 days (during the 5 days of use he received a dose of 375 mg/day) from September 2014 to July 2015, with no tumor growth until the beginning of 2016. After checking the evolution of the tumor by MRI in February 2016, there was an increase in the dimensions of the remaining lesion, notably in the temporal isthmus, which had a similar expansive effect on the adjacent encephalic structures. The patient was submitted to a partial surgical resection followed by chemoradiation and lasting 6 cycles of PCV associated with CBD. The CBD dosage was ranging from 100 to 200 mg/day.

During the chemoradiation he had an excellent clinical performance, practiced sports, and had few symptoms of fatigue and/or nausea.

**Figure 3:**
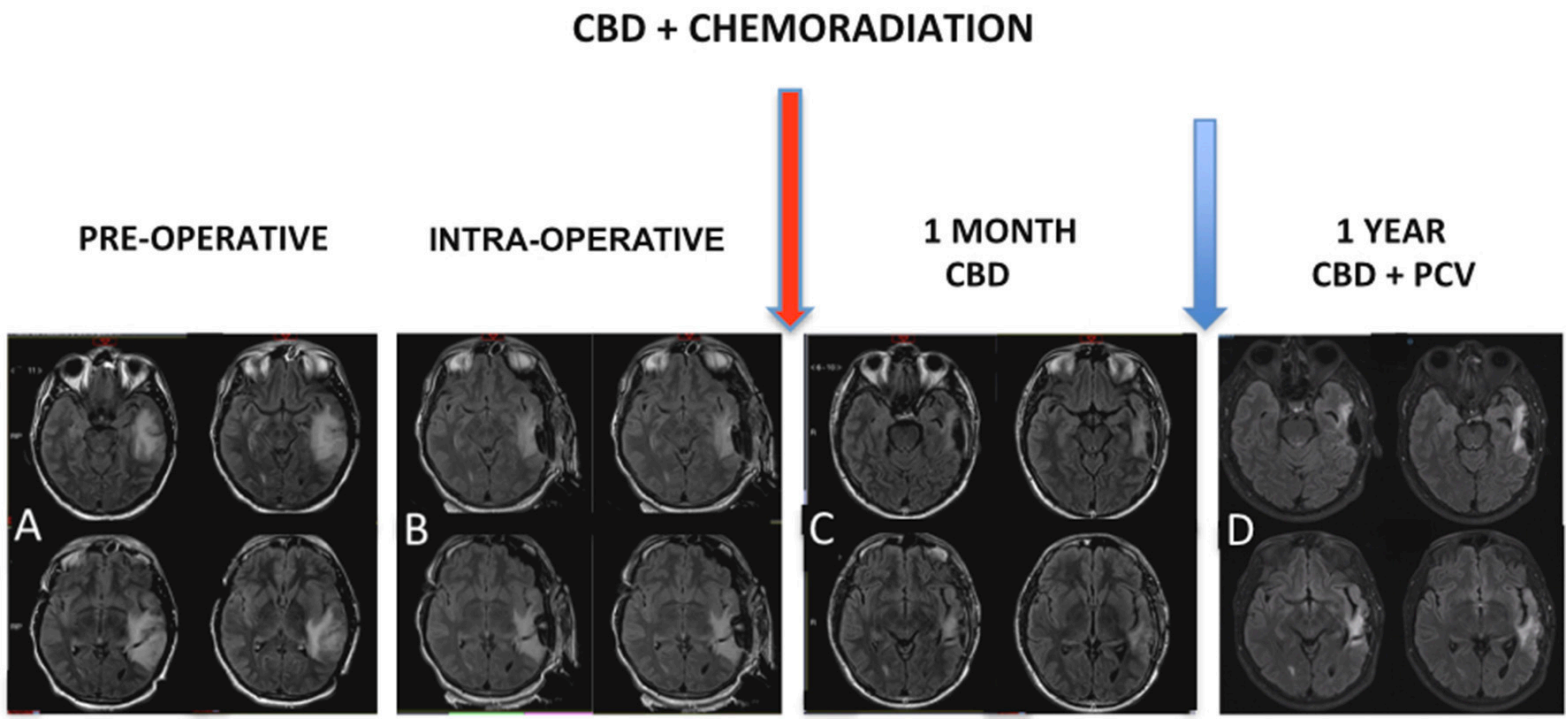
**(A)** MRI showed expansive infiltrative lesion predominantly subcortical of poorly defined contours, located in the left temporal lobe, involving the upper, middle and lower temporal gyrus, with increase of the left temporal gyrus cortex. The lesion compromises a large part of the temporal lobe and extends to the temporal isthmus, posterior aspect of the insula, and deeply to the trigeminal effigy of the left lateral ventricle. There is diffuse erasure of the regional cortical sulci and the Sylvian fissure, as well as slight compression over the atrium of the left lateral ventricle. **(B)** Intraoperative MRI. Red arrow: chemoradiation associated with cannabidiol in a dosage ranging from 100 to 200 mg/day. **(C)** MRI control right after chemoradiation was characterized post-operative changes associated with a significant reduction of the infiltrative components of the tumor. **(D)** Magnetic resonance control after 1 year characterized no evidence of disease progression.

**Figure 4:**
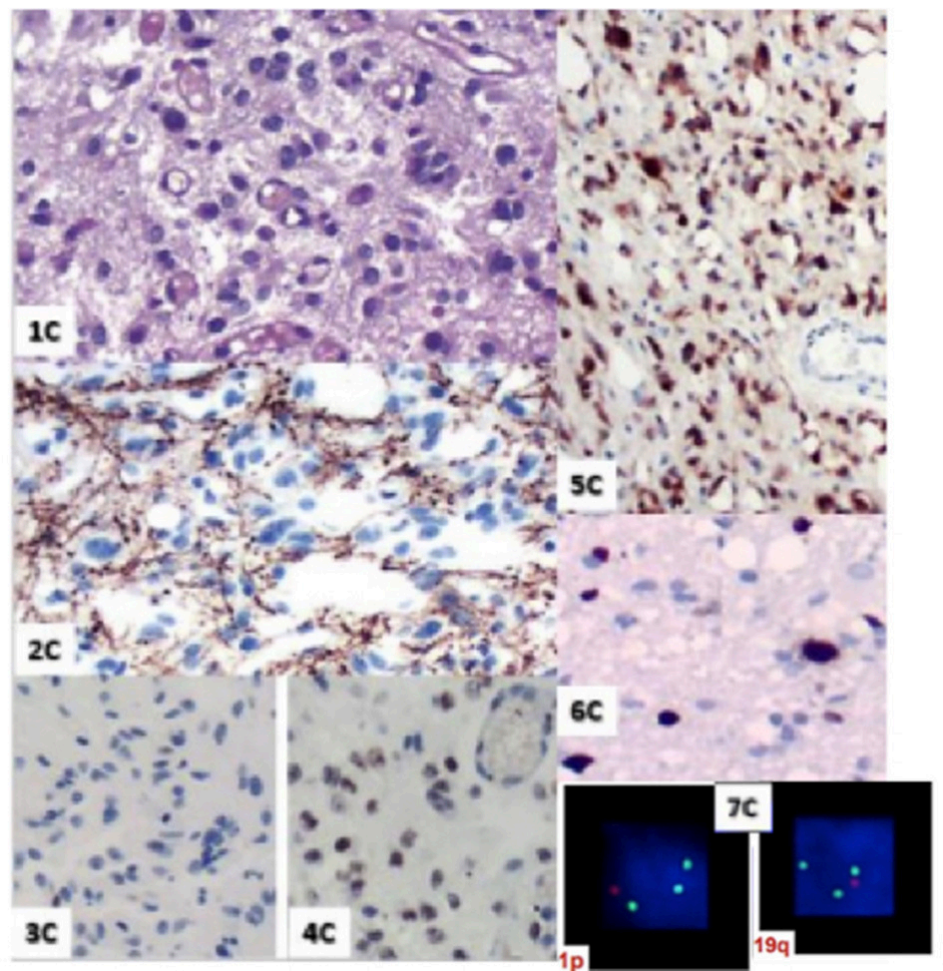
1 C. HE; 2C. GFAP; 3C. P53; 4C. Retained ATRX expression; 5C. IDH-mutated; 6C. Ki67: 9%; 7C. 1 p19q *I* Co-deleted.

MRI control immediately after chemoradiation (Figure 3) was used to characterize post-operative changes and showed a significant reduction of the infiltrative components of the tumor. The result of the pathological study after the first surgery (Figure 4) was oligodendroglioma grade II. After the second surgery, he was diagnosed as oligodendroglioma grade III characterized by an increase in Ki67 staining of 9% and increased cellularity. Biomolecular marker analysis indicated IDH-1 mutated and MGMT methylated.

## Discussion

Despite multimodal treatment, it is not possible to cure high-grade glioma patients. Therefore, the aim of treatment is not only to prolong life, but also to prevent deterioration of health-related quality of life as much as possible. In these two cases, we observed a significant improvement of clinical evolution. Both had a positive response to the treatment, with no evidence of disease progression for at least for 2 years and they are both alive. Unfortunately, the patient 1 presented tumor recurrence in the brainstem after approximately two and a half years of starting the treatment with chemoradiation followed by PCV.

Even with the prolonged use of CBD, the two patients did not develop any significant alterations in blood counts/plasma biochemistry, which is in accord with other studies showing that the prolonged use of CBD did not significantly affect hepatic or cardiac functions (24, 25). In contrast, a study evaluating the effectiveness and safety of CBD as an adjunctive treatment for seizures in patients with Lennox-Gastaut syndrome using meta-analytical techniques, observed increased alanine or aspartate aminotransferases more than three times the upper normal limit (14.5% vs. 0.6%, respectively) (26). It is important to note that the patients were using high doses of CBD (20 mg/kg/day) associated with anti-convulsive drugs.

The most common side effects associated with chemoradiation are chronic fatigue, loss of appetite, and nausea (27). In addition, the use of steroids causes side effects including increased appetite, agitation, insomnia, moon facies, fatigue, and myopathy (28). Both patients in the current study did not present with any of these side effects, nor did the usual continuous use of steroids during this phase of treatment. In addition, they could maintain their usual work and sports activities.

One of the patients presented with an exacerbated inflammatory response in the first MRI control soon after chemoradiation. The anti-inflammatory and neuroprotective actions of CBD may be related to the absence of side effects associated with PSD, such as headache, or changes relevant to tumor location (29, 30).

The high toxicity associated with PCV has an important impact on the course of treatment (31). A study stated that 28.5% of patients had to stop chemotherapy because of the severe side effects (32). Another study reported a delay in the treatment in 31.3% of patients to permit the toxicity to resolve (33). PCV chemotherapy is associated with major adverse events that need to be taken into consideration. Procarbazine, lomustine, and vincristine-induced hematological toxicity is severe as previous studies reported grade 3 lymphopenia and thrombocytopenia in 75 and 64% of patients, respectively (34). Another study reported that procarbazine induced major hepatotoxicity because it is metabolized by hepatic enzymes (35). Vincristine, as well as anticonvulsants, can also induce hepatic toxicity (33). Nausea and emesis were reported in 70–80% of patients receiving PCV without anti-nausea drugs (34, 36). Neurotoxicity, mostly attributable to vincristine, was also reported (37). Finally, rash was reported as a side effect of PCV (34, 36, 38).

The most common side effects of prolonged used of CBD are somnolence, decreased appetite, gastrointestinal disorders (diarrhea and nausea) (10–13) and increased transaminases levels (39, 40). In our two case studies, treatment with PCV associated with CBD did not cause lymphopenia, thrombocytopenia, hepatic toxicity, or neurotoxicity; however, a rash was observed in one patient and despite the fact that THC was often inhaled in the course of the PCV treatment, moderate nausea, emesis and fatigue were observed in both patients. No negative side effects were reported of the use of THC, but instead an increase in appetite and a reduction of fatigue were observed. The psychoactive effect of THC was considered positive as well as mood improving.

Studies of CBD in animal models of glioma reported its anti-tumor activity (41, 42). Preclinical studies support the idea that the combined administration of TMZ and cannabinoids might be therapeutically exploited for the management of GBM (43, 44). Results showed that the oral administration of THC and CBD in combination with TMZ produced a strong antitumoral effect in both subcutaneous and intracranial glioma cell-derived tumor xenografts (44). Another study investigated the effect of THC and CBD alone and in combination with radiotherapy in a number of glioma cell lines and in an orthotopic murine model for glioma. They showed dramatic reductions in tumor volumes when both cannabinoids were used with irradiation (45).

Two clinical studies (46, 47) of cannabinoid-based therapies in gliomas have been reported. Both clearly showed that cannabinoids did not facilitate tumor growth or decrease patient survival. A phase II clinical trial of 21 patients showed that those treated with a combination of THC and CBD in addition to TMZ had an 83% 1 year survival rate compared with 44% for those who did not receive the study drug. The median survival of the treated group was >662 days compared with 369 days in the group who did not receive the study drug (47). These first results of clinical investigations are promising and indicate the importance of cannabinoid translational research leading to clinically relevant studies.

Histologically, the presence of the 2 mutations, *1p19q* and *IDH1*, have been identified as factors with a favorable prognosis (48, 49), and their impact on the clinical course of glioma patients led to a change in the World Health Organization (WHO) classification in 2007 (6). Both of our patients had IDH-1 mutated and one patient had a 1p19q co-deletion, suggesting they are more likely to respond to the treatment and have a longer life expectancy.

The study had several limitations. Previous preclinical and clinical studies evaluated the combination of THC and CBD associated with TMZ. In this case report it was not possible to use TMZ because the patients had already failed this therapy. We could not find any study that described an association between treatment with PCV and cannabinoids. Furthermore, previous studies reported that combined THC and CBD treatment had a greater anti-tumor effect and impact on survival compared with THC or CBD alone. In this report, it was not possible to make such an association for legal reasons and the absence of a medication containing high doses of THC. Both patients were under 40 years old and had molecular markers that favored a better prognosis. Of note, they remained without disease progression during the time of the study, and they did not develop major side effects between the clinical course of chemoradiation to the follow-up of 6 cycles with PCV drugs, which often prevent the completion of treatment.

Although this study only had two cases, it is interesting to note the good clinical and radiological evolution that might be related to this therapeutic association. Future randomized placebo-controlled trials with a larger number of patients are needed to confirm the study findings.

The wide use of CBD in the neuro-oncology field should be undertaken with caution. Preclinical and clinical studies are essential to demonstrate interactions with standard of care treatments, and its effects on the symptoms, quality of life, and possible immunomodulation should be determined.

These observations are of particular interest because the pharmacology of cannabinoids appears to be distinct from existing oncology medications and may offer a unique and possibly synergistic option for future glioma treatment.

## Ethics Statement

Patients submitted for treatment with cannabinoids had to complete an extensive form. This form was then sent to Anvisa, Brazil's highest health regulatory agency for approval. Therefore, permission by the local ethics committee was not required. The patients signed consent forms, which are attached to the medical records.

## Author Contributions

PD contributed to the design and implementation of the cannabidiol in the study and to the writing of the manuscript. MM and OF were involved in planning and supervised the study. MD brilliantly interpreted the radiological images and the follow-up and with CL and PD designed the figures. CL performed the pathology's diagnostic and the complete analysis of the surgery material. Also CL encourage PD to investigate the use of cannabidiol in neuro-oncology tumours and supervised the findings of this work.

### Conflict of Interest Statement

The authors declare that the research was conducted in the absence of any commercial or financial relationships that could be construed as a potential conflict of interest.
